# Machine Learning Approach to Analyze the Heavy Quark Diffusion Coefficient in Relativistic Heavy Ion Collisions

**DOI:** 10.3390/e25111563

**Published:** 2023-11-20

**Authors:** Rui Guo, Yonghui Li, Baoyi Chen

**Affiliations:** 1 Data Science, Washington University, St. Louis, MO 63105, USA; grui@wustl.edu; 2 Department of Physics, Tianjin University, Tianjin 300354, China

**Keywords:** quark-gluon plasma, relativistic heavy-ion collisions, heavy quark, machine learning, diffusion coefficient

## Abstract

The diffusion coefficient of heavy quarks in a deconfined medium is examined in this research using a deep convolutional neural network (CNN) that is trained with data from relativistic heavy ion collisions involving heavy flavor hadrons. The CNN is trained using observables such as the nuclear modification factor $R_{AA}$ and the elliptic flow $v_{2}$ of non-prompt J/ψ from the B-hadron decay in different centralities, where B meson evolutions are calculated using the Langevin equation and the instantaneous coalescence model. The CNN outputs the parameters, thereby characterizing the temperature and momentum dependence of the heavy quark diffusion coefficient. By inputting the experimental data of the non-prompt J/ψ$\left(R_{AA},v_{2}\right)$ from various collision centralities into multiple channels of a well-trained network, we derive the values of the diffusion coefficient parameters. Additionally, we evaluate the uncertainty in determining the diffusion coefficient by taking into account the uncertainties present in the experimental data $\left(R_{AA},v_{2}\right)$, which serve as inputs to the deep neural network.

## 1. Introduction

In recent years, there has been rapid development in deep learning methods, which are increasingly being widely applied in industry and scientific research. In particular, deep learning methods are being used to handle high-dimensional data and to uncover patterns, such as in image recognition. In the realm of scientific research, deep learning has already found applications in many aspects of physics [1,2,3]. In the theoretical research of high-energy nuclear physics, an increasing number of studies are utilizing deep learning methods to analyze the computational data from theoretical models, such as the study of the equation of the state of QGP matter [4], the dynamical evolutions of QGP [5] and identifications of the spinodal clumping in high-energy nuclear collisions [6].

In the Relativistic Heavy-Ion Collider (RHIC) and the Large Hadron Collider (LHC) [7,8,9], a new kind of deconfined state, called the Quark-Gluon Plasma (QGP), was predicted and produced. Properties such as the initial energy density and coupling strength of QGP have been typically studied through the final-state light hadron spectra [10,11,12,13] or through the distribution of heavy flavor particles [14,15,16,17,18,19]. For heavy flavor particles, their distribution is initially influenced by the cold nuclear effect, and then they will interact with the QGP medium, thus resulting in energy loss [20,21,22,23]. Numerous models have been established to account for the various effects mentioned above [24,25,26,27,28,29], whereby the aim is to ultimately predict the nuclear modification factor and the anisotropic flows of open heavy flavor hadrons. These models have been used to investigate the energy loss mechanisms of heavy quarks in the QGP from different perspectives. Relevant nuclear modification factors and the collective flows of D mesons or B mesons have also been experimentally measured by STAR [30], ALICE [31,32,33] and CMS [34,35] collaborations, which are closely related to the energy loss process of heavy quarks. As it is not straightforward to explain the experimental observables of $R_{AA}$ and $v_{2}\left(p_{T}\right)$ at the same time when using a simple value for the diffusion coefficient $D_{s}2\pi T$, a more realistic expression with temperature and momentum dependence is needed. Due to the complex process involving the heavy quark diffusion and hydrodynamic evolution, it is necessary to employ deep neural networks to analyze the relationship between the diffusion coefficient and experimental observables. CNN has been proven to be suitable for analyzing high-dimensional datasets and for quantifying the value of a diffusion coefficient when considering multiple hot and cold nuclear matter effects.

In previous studies, Bayesian statistical analysis has been employed to analyze the experimental data of soft particles [36,37,38,39], whereby the diffusion coefficient is quantitatively extracted with experimental observables of the charmed hadrons in heavy-ion collisions [40]. Although there is abundant experimental data on D mesons, our main focus is on studying the evolution of B mesons. This is because the diffusion coefficient is directly related to the drag term in the Langevin equation, which arises from elastic scattering processes rather than medium-induced gluon radiation. Therefore, as the mass of a heavy quark increases, the contribution from elastic scattering processes becomes more significant in the energy loss process, while the contribution from gluon radiation is relatively weaker. In this work, we treat the $R_{AA}$ and $v_{2}$ of non-prompt J/ψ from the B meson decay as inputs of the CNN, and the parameters in the diffusion coefficient are treated as outputs of the network. After training the neural network with supervision, the inputs of the neural network are selected from values within the error bars of the experimental data points in order to generate the corresponding diffusion coefficient (albeit with some uncertainty).

This paper is organized as follows: in Section 2, we introduce the Langevin plus instantaneous coalescence model (LICM) to generate datasets of heavy flavor evolutions that had different values in terms of their parameters (which were then used as training datasets for the CNN model). In Section 3, the values of the shadowing factor, temperature and momentum dependence of the diffusion coefficient are quantitatively extracted based on the experimental data of the non-prompt J/ψ from B meson decay. A final summary is given in Section 4.

## 2. Frameworks

### 2.1. Generating Datasets with LICM

Bottom quarks are produced in the hard scatterings of nucleon partons. We adopted the fixed-order plus next-to-leading log formula (FONLL) [41,42], and the NNPDF30NLO PDF set [43] was used to calculated the initial momentum distribution of the bottom quarks in nucleon–nucleon collisions. Due to the fact that Pb–Pb collisions can be regarded as a superposition of nucleon–nucleon collisions, which are accompanied by cold nuclear matter effects, the initial momentum distribution of the bottom quarks in Pb–Pb collisions can be considered as the momentum distribution in the pp collisions that are multiplied by a shadowing factor. The nuclear shadowing factor was calculated with the EPS09 NLO package [44] in 5.02 TeV Pb-Pb collisions. The production of the bottom quarks primarily arose from binary collision processes; hence, the spatial distribution of the bottom quarks in nuclear collisions was proportional to the distribution of binary collisions $dN_{b\bar{b}}/dx_{T}\propto T_{A}\left(x_{T}-b/2\right)T_{B}\left(x_{T}+b/2\right)$ [45]. Here, $T_{A(B)}=\int dz\rho_{A(B)}\left(x_{T},z\right)$ represents the thickness functions of the two nuclei. The nucleon distribution $\rho (x_{T},z)$ in the nucleus follows a Woods–Saxon distribution.

After the production of bottom quarks, they propagate within the high-temperature QGP medium and are accompanied by energy loss. The energy loss of bottom quarks in the QGP is primarily attributed to the scattering processes between bottom quarks and thermal partons, as well as medium-induced parton radiation. Considering that the mass of the bottom quark is much larger than the typical temperature of the medium, the momentum change during each interaction of the bottom quarks in the medium is relatively small, and it can be regarded as Brownian motion. Consequently, the momentum evolution of bottom quarks can be described using the Langevin equation:

(1) $$\begin{array}{c} \frac{dp}{dt}=-\eta_{D}p+\xi +f_{g}. \end{array}$$

On the right-hand side, the first two terms represent the drag and noise terms, which come from the elastic collisions with thermal light partons. The drag coefficient is defined as $\eta_{D}\left(p\right)=\kappa /\left(2TE_{b}\right)$, where the energy of the bottom quark is given by $E_{b}=\sqrt{m_{b}^{2}+p^{2}}$. The mass of the bottom quark is taken to be $m_{b}=4.75$ GeV. κ represents the momentum–diffusion coefficient. It is related to the spatial diffusion coefficient $D_{s}$ via $D_{s}\kappa =2T^{2}$. In the noise term ξ, the time correlation and momentum dependence are both neglected for simplicity, where ξ is treated as a Gaussian-shaped white noise that satisfies the following relation:

(2) $$\begin{array}{c} \langle \xi^{i}\left(t\right)\xi^{j}\left(t^{\prime }\right)\rangle =\kappa \delta^{ij}\delta \left(t-t^{\prime }\right). \end{array}$$

The third term $f_{g}=-dp_{g}/dt$, with $p_{g}$ being the momentum of the emitted gluon, represents the recoil force on the bottom quark from the emitted gluon. The number of emitted gluons in a small time interval t∼t+Δt is [46] are represented by

(3) $$\begin{array}{c} P_{\mathrm{rad}}\left(t,\Delta t\right)=\langle N_{g}\left(t,\Delta t\right)\rangle =\Delta t\int dxdk_{T}^{2}\frac{dN_{g}}{dxdk_{T}^{2}dt}. \end{array}$$

Here, $x=E_{g}/E_{b}$ represents the ratio of the energy carried by the gluons that are radiated from the bottom quarks. $dN_{g}/dxdk_{T}^{2}dt$ is the the spectrum of an emitted gluon from a higher twist calculation [22,23]. $k_{T}$ is the transverse momentum of the gluon. The position of a heavy quark is updated in each time step as $x\left(t+\Delta t\right)=x\left(t\right)+p/E_{b}\cdot \Delta t$.

Heavy quarks are randomly generated based on the initial spatial and momentum distributions, and they propagate in the QGP with an energy loss that is described by Equation (1). When bottom quarks diffuse and move into certain regions, wherein the QGP local temperature is low, heavy quarks undergo hadronization by combining with light quarks to form B mesons, or by combining with an anti-heavy quark to form quarkonium. In the high-momentum region, the production of B mesons from bottom quarks is predominantly through fragmentation processes, while in the intermediate- and low-momentum regions, it is mainly through the coalescence process. In this study, we primarily focused on bottom quarks with a transverse momentum of $p_{T}\leq 15$ GeV/c. Therefore, we utilized a coalescence model to describe the hadronization process of bottom quarks into B mesons as follows:

(4) $$\begin{array}{c} \frac{dN_{M}}{dp_{M}}=\int \frac{dp_{1}}{{\left(2\pi \right)}^{3}}\frac{dp_{2}}{{\left(2\pi \right)}^{3}}\frac{dN_{1}}{dp_{1}}\frac{dN_{2}}{dp_{2}}f_{M}^{W}\left(q_{r}\right)\delta^{\left(3\right)}\left(p_{M}-p_{1}-p_{2}\right), \end{array}$$

The momentum distribution of the B meson $dN_{M}/dp_{M}$ is proportional to the distributions of the bottom quarks $dN_{1}/dp_{1}$ and also to the thermal light quarks $dN_{2}/dp_{2}$. The heavy quark distribution is given by the Langevin equation, while thermal light quarks are taken as a Fermi distribution. Their coalescence probability is determined by the Wigner function $f_{M}^{W}\left(q_{r}\right)$, which can be obtained via the Weyl transform of the B meson wave function. In principle, the complete Wigner function $f^{W}\left(q_{r},x_{r}\right)$ provides constraints on the relative distance and relative momentum that occurs between two particles in the formation of a bound state. The spatial constraint becomes crucial and can significantly reduce the coalescence probability when the two particles are rare in the QGP, as observed in the case of the charmonium coalescence process. However, in the case of a B meson composed of one heavy and one light anti-quark, with a plentiful number of light quarks in the QGP, we assume that the heavy quark can readily find a light quark in proximity, thus satisfying the spatial constraint. By integrating over the spatial part of the complete Wigner function, we simplify it to a normalized Gaussian function $A_{0}\exp\left(-q_{r}^{2}\sigma^{2}\right)$, where $A_{0}$ represents the normalization factor. The width of the Gaussian function is connected with the root–mean–square radius of the B meson $\sigma^{2}=\frac{4}{3}\frac{{\left(m_{1}+m_{2}\right)}^{2}}{m_{1}^{2}+m_{2}^{2}}{\langle r^{2}\rangle }_{B}$ [47], where the value is to be $\sqrt{{\langle r^{2}\rangle }_{B}}=0.43$ fm [45]. $m_{1}$ is the bottom quark mass, while the thermal mass of the light quark is approximated to be $m_{2}=0.3$ GeV, which is used in the coalescence process. $q_{r}=\left(E_{2}^{\mathrm{cm}}p_{1}^{\mathrm{cm}}-E_{1}^{\mathrm{cm}}p_{2}^{\mathrm{cm}}\right)/\left(E_{1}^{\mathrm{cm}}+E_{2}^{\mathrm{cm}}\right)$ is the relative momentum between the bottom quark and the light quark in the center of mass (COM) frame. $p_{1}^{\mathrm{cm}}$ and $p_{2}^{\mathrm{cm}}$ are the momenta of the bottom quark and the light quark in the COM frame. The delta function ensures the momentum conservation in the coalescence process $p_{M}=p_{1}+p_{2}$. As the phase transition between QGP and hadronic gas crossover at LHC energies, we performed the coalescence process at the critical temperature $T_{c}=150$ MeV. The time and spatial evolutions of bulk media have been well described with hydrodynamic equations. We employed the MUSIC package to provide the information for the hot medium at 5.02 TeV Pb–Pb collisions [27,48,49]. The local temperatures of the medium varied with coordinates and time, and these variations were then incorporated into the Langevin equation. After hadronization, the B mesons continue their diffusion in the hadronic gas with a different value of the diffusion coefficient, and they then decay into a non-prompt J/ψ after a kinetic freeze out at a temperature of $T_{\mathrm{fo}}=120$ MeV.

Recently, through lattice QCD calculations, new calculations of the spatial diffusion coefficient of heavy quarks at different temperatures have been presented [20], and they were found to be smaller than the previous quenched lattice QCD [50,51] and recent phenomenological estimates [40,52,53,54]. This conclusion was also observed in other theoretical results [55,56,57]. This prompted us to re-examine the relationship between the experimental measurements of heavy quarks and the diffusion coefficient. In high temperature and momentum regimes, the diffusion coefficient can be calculated through perturbative QCD [58,59,60]. However, this calculation is not sufficient for simultaneously explaining the $R_{AA}$ and $v_{2}$ of open heavy flavor particles that are measured in nuclear collisions [61], thus suggesting that non-perturbative processes play an indispensable role in the temperature and momentum dependence of the diffusion coefficient. In Bayesian statistical analysis, a parameterized form of the diffusion coefficient is proposed, including a linear temperature dependence term and a perturbative QCD term such as $D_{s}2\pi T\propto A\left(p\right)\left(\alpha +\beta T\right)+\left(1-A\left(p\right)\right)8\pi /\left(\hat{q}/T^{3}\right)$ [40]. The first term represents the contribution from non-perturbative processes, while the second term represents the contribution from perturbative processes. $\hat{q}$ is the heavy quark transport coefficient, which is calculated by the elastic scatterings between heavy and light quarks [62]. The spatial diffusion coefficient from lattice QCD calculations was found in the p=0 case. In this work, we introduce the following concise formula to consider the temperature and momentum dependence of the spatial diffusion coefficient:

(5) $$\begin{array}{c} D_{s}2\pi T=\left[\alpha +\beta \left(\frac{T}{T_{c}}-1\right)\right]\times {\left(\frac{m_{Q}}{E_{Q}}\right)}^{\gamma }. \end{array}$$

The temperature and momentum dependence were encoded in the terms $T/T_{c}$ and $m_{Q}/E_{Q}$, where $m_{Q}$ and $E_{Q}=\sqrt{m_{Q}^{2}+p_{Q}^{2}}$ are the mass and energy of heavy quarks, respectively. The parameter α represents the value of $D_{s}2\pi T$ at a critical temperature, where the momentum is p=0. The parameters β and γ control the degree of temperature and momentum dependence. In hadronic gas, the coupling strength between B and D mesons with the medium becomes much smaller. Their contribution on the $R_{AA}$ and $v_{2}$ of open heavy flavor particles are limited. In hadronic gas, the mean value of the spatial diffusion coefficient of B mesons is approximated to be $D_{s}^{M}2\pi T=9$ before the kinetic freeze out of B mesons in $0.8T_{c}<T<T_{c}$ [63].

### 2.2. Deep Neural Networks

**Figure 1:**
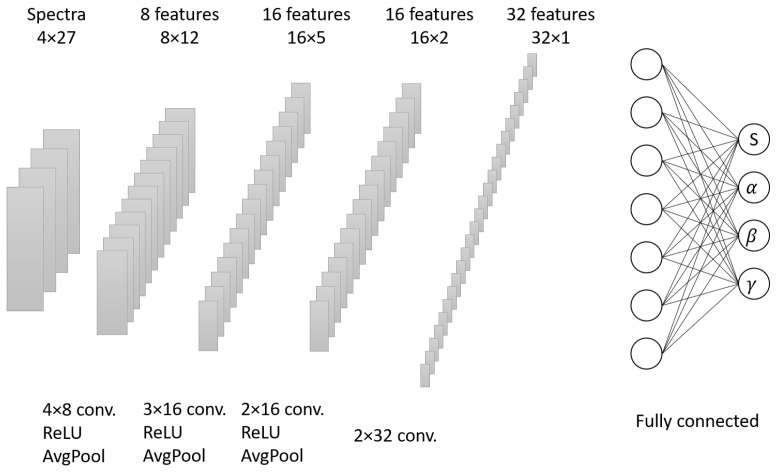
Schematic figure that shows the structure of the convolutional neural network. The $R_{AA}\left(p_{T}\right)$ in the three centralities and the one $v_{2}\left(p_{T}\right)$ are taken as four channels of the input layer, while the parameters related to the spatial diffusion coefficient are the output.

In the dynamical evolution of bottom quarks, our theoretical model produces a wide range of $R_{AA}$ and $v_{2}$ values for the non-prompt J/ψ in 5.02 TeV Pb–Pb collisions by varying the shadowing factor (which is denoted as *S*) and the three parameters of (α,β,γ) in Equation (5). This dataset will serve as the training data for the CNN. Experimental measurements were conducted to determine the nuclear modification factor $R_{AA}\left(p_{T}\right)$ for the non-prompt J/ψ in three different centralities, as well as the elliptic flow coefficient of $v_{2}\left(p_{T}\right)$. In our approach, we treat the three centralities of $R_{AA}\left(p_{T}\right)$ as separate channels, while $v_{2}\left(p_{T}\right)$ acts as an additional channel in the input layer of the CNN. The output layer of the CNN incorporates the corresponding parameter values in the diffusion coefficient and the shadowing factor, which are considered labels for the input data. Theoretical calculations based on the LICM model establish a mapping relationship between the parameter combination values (S,α,β,γ) and the experimental observables $\left(R_{AA},v_{2}\right)$. Figure 1 provides a graphical representation of the network structure, which consists of four hidden layers, including the average pooling layers and fully connected layers. The ReLU activation function was chosen for these layers. The three output channels related to the diffusion coefficient parameters were projected to have positive values, while the channel associated with the shadowing effect was projected within the range of 0–1 when using the Sigmoid function.

To generate the training dataset, we randomly selected parameter values within the regions specified in Table 1 using the Langevin model. Due to the significant uncertainty surrounding the shadowing factor *S* in the model calculations, which can notably impact the final observables of the B meson, we considered the shadowing factor as a parameter to be optimized within the deep neural network. The values of the shadowing factor were constrained within the range of 0.6 to 1.0. For the 5.02 TeV Pb–Pb collisions, the values of *S* used to generate the training dataset were randomly selected from the range of 0.6 to 1.0. The spatial diffusion coefficient $D_{s}2\pi T$ at the critical temperature of $T_{c}$ was selected within the range of 2.0≤α≤6.0, while the values of β and γ that characterized the temperature and momentum dependence were chosen within the ranges of 0≤β≤8.0 and 0≤γ≤1.0, respectively. We generated 20 K events as the training and validation datasets, where each event corresponded to one of the combinations of the parameters. The performance of the neural network was influenced by both the size of the training dataset and the structure of the deep neural network. This type of uncertainty has been examined in previous studies [64], and it will be partially investigated in this work by varying the size of the training datasets. However, such uncertainties were not as significant as those arising from the error bars of the experimental data points of heavy flavor particles that were used as the input for the CNN.

## 3. Results and Analyses

**Figure 2:**
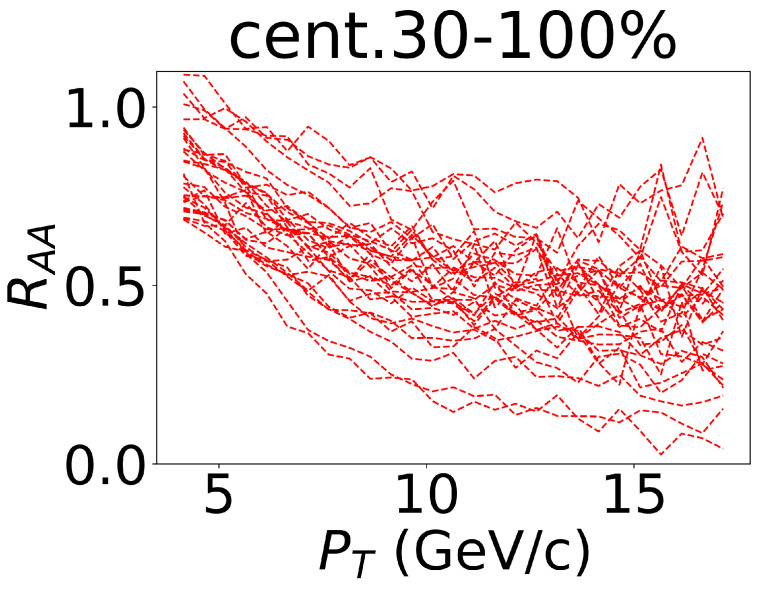
Some of the events that were randomly selected from one channel of the training dataset. The lines represent the nuclear modification factors of the non-prompt J/ψ, which was calculated with different parameter values in the centrality of 30–100% in the 5.02 TeV Pb–Pb collisions.

In the previous sections, we introduced the theoretical model to generate the training dataset for the CNN by varying the values of the parameters in the model. We plotted some events ($\left(R_{AA}\left(p_{T}\right),v_{2}\left(p_{T}\right)\right)$), and these were randomly selected from one channel of the training datasets, as shown in Figure 2. The lines could cover the experimental data, which indicated that the training range of the model encompassed the distribution of the experimental data. The model was able to be effectively applied to analyze the experimental data. As the experimental data points about $R_{AA}$ were located in $p_{T}\geq 2$ GeV/c, and as $v_{2}$ was located in $p_{T}\geq 4$ GeV/c, we truncated the training data by only retaining the data with $p_{T}$ values above 4 GeV/c. This ensured that the shape of the training data aligned as closely as possible with the experimental data, thus making it easier to incorporate them into the input (please see Figure 2).

**Figure 3:**
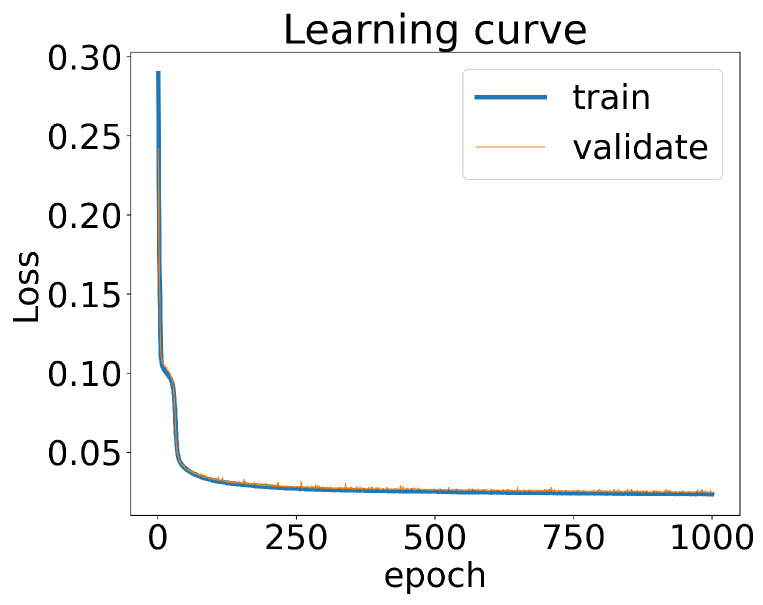
The loss of the CNN model as a function of the training epochs. The loss of the model calculated with the training datasets and the validation datasets are respectively plotted.

We partitioned 70% of the total datasets as the training data, while the remaining 30% of the datasets served as the validation data. By treating $\left(R_{AA},v_{2}\right)$ as the inputs for the CNN and the parameter values as labels, we could calculate the loss of the CNN for both the training and validation datasets. The learning curve shown in Figure 3 demonstrates that the loss decreased to below 5% after 250 training epochs. Notably, the loss of the CNN for the training datasets closely aligned with the loss observed when using the validation datasets. This indicated that the CNN model did not exhibit significant overfitting or underfitting.

**Figure 4:**
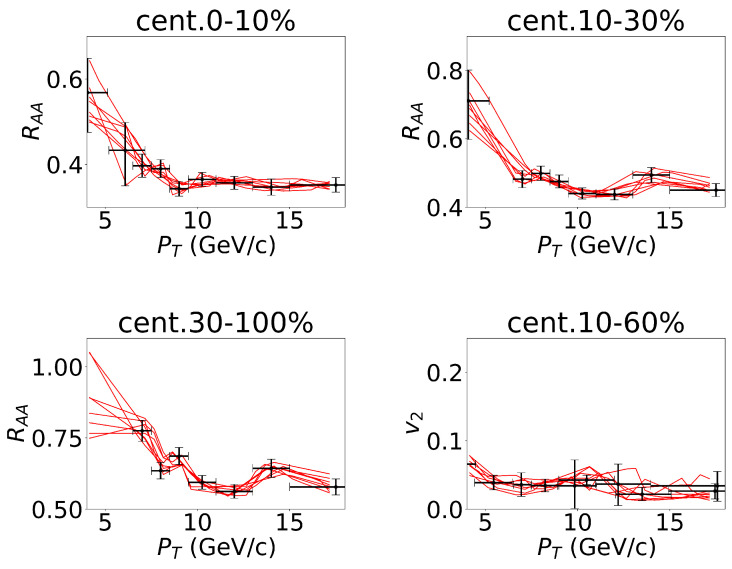
To consider the error bars of the experimental data about non-prompt J/ψ in the 5.02 TeV Pb–Pb collisions, we sampled the values of $R_{AA}$ and $v_{2}$ within the error bars of each data point, and we took them as inputs of the deep neural network. Some of the lines representing random events are plotted in the figures. The four figures represent the four channels of the network. The experimental data were cited from CMS Collaboration [34,35].

In using the well-trained CNN model, we fed the experimental data points into the neural network. Considering the presence of error bars in the experimental data points (as depicted in Figure 4), we sampled within the error bars associated with the experimental data points. These samples were then utilized as inputs to the neural network, thus allowing us to account for the impact of experimental uncertainties on the diffusion coefficient parameters. To capture the influence of the experimental uncertainty, we randomly generated 10 K samples within the range of experimental error bars around the data points, as illustrated in Figure 4.

**Figure 5:**
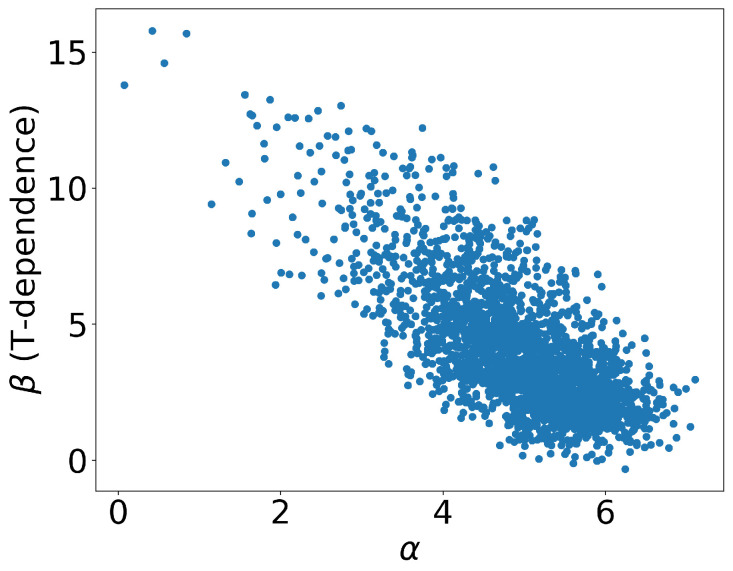
The distribution of the parameter values (α,β) from the CNN. The x-axis represents α, while the y-axis represents β. Each point represents one event.

**Figure 6:**
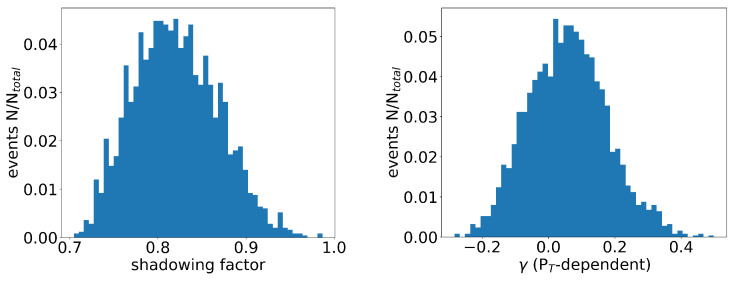
The distribution of the parameter values (S,γ) obtained from the CNN model is shown in the plot. The x-axis corresponds to the values of *S* or γ, while the y-axis represents the number of events.

Consequently, we obtained 10 K different outputs, which are plotted in Figure 5. Each data point represents an individual event, with the corresponding values of α and β represented on the x- and y-axes, respectively. As previously mentioned, α signifies the value of $D_{s}2\pi T$ at critical temperature and zero momentum, while β represents its temperature dependence. The majority of events were concentrated within the region of 4≤α≤6.5 and 0≤β≤5.0. Furthermore, we also plotted the values of γ, which helped with characterizing the transverse momentum dependence, as well as the shadowing factor *S* that arises from the cold nuclear matter effect shown in Figure 6. From the distributions, it was observed that the majority of the events fell within the range of 0.0≤γ≤0.2 and 0.75≤S≤0.9, as depicted in Figure 6. The distribution of the events in the figure reflected the uncertainty in the parameter values that resulted from the experimental data errors, as well as the neural network structure.

Based on the distributions obtained from the CNN outputs, we can directly extract the mean values of these parameters, which are presented in Table 2. It is worth noting that the value of α remains larger than the results obtained from the lattice QCD calculations at $T=T_{c}$ [20], and these are consistent with the values given by previous model calculations. Additionally, the temperature dependence (β) was found to be strong, while the momentum dependence (γ) was relatively weak.

## 4. Summary

In this study, a convolutional neural network was employed to extract the temperature and momentum dependence in the spatial diffusion coefficient of heavy quarks with the experimental data obtained from the non-prompt J/ψ decays in B mesons. The Langevin equation was utilized to describe the dynamical evolution of the heavy quarks in the QGP and the B mesons in the hadronic gas. Additionally, the instantaneous coalescence model was used to describe the hadronization process from the bottom quarks to the B mesons. By taking different values for the shadowing factor and diffusion coefficient, the nuclear modification factors and elliptic flows of the non-prompt J/ψ in multiple centralities of 5.02 TeV Pb–Pb collisions were generated. The CNN model was trained under supervision with model calculations. To extract the values of the diffusion coefficient, we sampled $\left(R_{AA},v_{2}\right)$ from the experimental data points along with their error bars, which were then used as inputs for the CNN. By doing so, the corresponding values of the diffusion coefficient and shadowing factor were obtained concurrently. The dispersion in the diffusion coefficient values can be partially attributed to the uncertainties present in the experimental data. The mean values of the diffusion coefficient were also extracted. This research contributes to the understanding of the heavy quark diffusion coefficient through a data-driven analysis approach.

## Figures and Tables

**Table 1:** The samples of the parameters used in the training dataset.

Parameters	Region
Shadow factor	S∈[0.6,1.0]
$D_{s}2\pi T$	α∈[2.0,6.0]
with	
T-dependence	β∈[0.0,8.0]
$p_{T}$-dependence	γ∈[0.0,1.0]

**Table 2:** The mean values and the standard deviation of the parameters were extracted using the experimental data points for the non-prompt J/ψ$R_{AA}\left(p_{T}\right)$ and $v_{2}\left(p_{T}\right)$ in the 5.02 TeV Pb–Pb Collisions.

Parameters	Mean Values	Standard Deviation
Shadow factor	〈S〉=0.82	0.050
$D_{s}2\pi T$	〈α〉=4.87	0.90
with		
T-dependence	〈β〉=4.16	2.32
$p_{T}$-dependence	〈γ〉=0.058	0.12

## Data Availability

Data available on request due to restrictions eg privacy or ethical The data presented in this study are available on request from the corresponding author. The data are not publicly available due to data storage issues.

## References

[1] Radovic A., Williams M., Rousseau D., Kagan M., Bonacorsi D., Himmel A., Aurisano A., Terao K., Wongjirad T. (2018). Machine learning at the energy and intensity frontiers of particle physics. Nature.

[2] Carleo G., Cirac I., Cranmer K., Daudet L., Schuld M., Tishby N., Vogt-Maranto L., Zdeborová L. (2019). Machine learning and the physical sciences. Rev. Mod. Phys..

[3] Mehta P., Bukov M., Wang C.H., Day A.G.R., Richardson C., Fisher C.K., Schwab D.J. (2019). A high-bias, low-variance introduction to Machine Learning for physicists. Phys. Rept..

[4] Pang L.G., Zhou K., Su N., Petersen H., Stöcker H., Wang X.N. (2018). An equation-of-state-meter of quantum chromodynamics transition from deep learning. Nat.Commun..

[5] Huang H., Xiao B., Liu Z., Wu Z., Mu Y., Song H. (2021). Applications of deep learning to relativistic hydrodynamics. Phys. Rev. Res..

[6] Steinheimer J., Pang L., Zhou K., Koch V., Randrup J., Stoecker H. (2019). A machine learning study to identify spinodal clumping in high energy nuclear collisions. J. High Energy Phys..

[7] Aoki Y., Endrodi G., Fodor Z., Katz S.D., Szabo K.K. (2006). The Order of the quantum chromodynamics transition predicted by the standard model of particle physics. Nature.

[8] Bazavov A., Bhattacharya T., Cheng M., DeTar C., Ding H.T., Gottlieb S., Gupta R., Hegde P., Heller U.M., Karsch F. (2012). The chiral and deconfinement aspects of the QCD transition. Phys. Rev. D.

[9] Shuryak E.V. (1980). Quantum Chromodynamics and the Theory of Superdense Matter. Phys. Rept..

[10] Afanasiev S.V., Anticic T., Barna D., Bartke J., Barton R.A., Behler M., Betev L., Bialkowska H., Billmeier A., Blume C. (2002). Energy dependence of pion and kaon production in central Pb + Pb collisions. Phys. Rev. C.

[11] Song H., Heinz U.W. (2008). Causal viscous hydrodynamics in 2+1 dimensions for relativistic heavy-ion collisions. Phys. Rev. C.

[12] Song H., Bass S.A., Heinz U., Hirano T., Shen C. (2011). 200 A GeV Au+Au collisions serve a nearly perfect quark-gluon liquid. Phys. Rev. Lett..

[13] Shen C., Heinz U., Huovinen P., Song H. (2011). Radial and elliptic flow in Pb+Pb collisions at the Large Hadron Collider from viscous hydrodynamic. Phys. Rev. C.

[14] Matsui T., Satz H. (1986). *J*/*ψ* Suppression by Quark-Gluon Plasma Formation. Phys. Lett. B.

[15] Andronic A., Braun-Munzinger P., Redlich K., Stachel J. (2003). Statistical hadronization of charm in heavy ion collisions at SPS, RHIC and LHC. Phys. Lett. B.

[16] Rapp R., Blaschke D., Crochet P. (2010). Charmonium and bottomonium production in heavy-ion collisions. Prog. Part. Nucl. Phys..

[17] Qin G.Y., Wang X.N. (2015). Jet quenching in high-energy heavy-ion collisions. Int. J. Mod. Phys. E.

[18] Liu Y., Chen B., Xu N., Zhuang P. (2011). Υ Production as a Probe for Early State Dynamics in High Energy Nuclear Collisions at RHIC. Phys. Lett. B.

[19] Yan L., Zhuang P., Xu N. (2006). Competition between J/*ψ* suppression and regeneration in quark-gluon plasma. Phys. Rev. Lett..

[20] Altenkort L., Kaczmarek O., Larsen R., Mukherjee S., Petreczky P., Shu H.-T., Stendebach S. (2023). Heavy Quark Diffusion from 2+1 Flavor Lattice QCD with 320 MeV Pion Mass. Phys. Rev. Lett..

[21] Qin G.Y., Ruppert J., Gale C., Jeon S., Moore G.D., Mustafa M.G. (2008). Radiative and collisional jet energy loss in the quark-gluon plasma at RHIC. Phys. Rev. Lett..

[22] Guo X.F., Wang X.N. (2000). Multiple scattering, parton energy loss and modified fragmentation functions in deeply inelastic e A scattering. Phys. Rev. Lett..

[23] Zhang B.W., Wang E., Wang X.N. (2004). Heavy quark energy loss in nuclear medium. Phys. Rev. Lett..

[24] He M., Fries R.J., Rapp R. (2014). Heavy Flavor at the Large Hadron Collider in a Strong Coupling Approach. Phys. Lett. B.

[25] Cao S., Luo T., Qin G.Y., Wang X.N. (2016). Linearized Boltzmann transport model for jet propagation in the quark-gluon plasma: Heavy quark evolution. Phys. Rev. C.

[26] Ke W., Xu Y., Bass S.A. (2019). Modified Boltzmann approach for modeling the splitting vertices induced by the hot QCD medium in the deep Landau-Pomeranchuk-Migdal region. Phys. Rev. C.

[27] Chen B., Jiang L., Liu X.H., Liu Y., Zhao J. (2022). X(3872) production in relativistic heavy-ion collisions. Phys. Rev. C.

[28] Chen B., Zhao J. (2017). Bottomonium Continuous Production from Unequilibrium Bottom Quarks in Ultrarelativistic Heavy Ion Collisions. Phys. Lett. B.

[29] Akamatsu Y., Asakawa M., Kajimoto S., Rothkopf A. (2018). Quantum dissipation of a heavy quark from a nonlinear stochastic Schrödinger equation. J. High Energy Phys..

[30] Adamczyk L., Adkins J.K., Agakishiev G., Aggarwal M.M., Ahammed Z., Ajitanand N.N., Alekseev I., Anderson D.M., Aoyama R., Aparin A. (2017). Measurement of *D*^0^ Azimuthal Anisotropy at Midrapidity in Au+Au Collisions at $\sqrt{s_{\mathrm{NN}}}$ = 200 GeV. Phys. Rev. Lett..

[31] Abelev B., Adam J., Adamová D., Adare A.M., Aggarwal M.M., Rinella G.A., Agnello M., Agocs A.G., Agostinelli A., Ahammed Z. (2013). D meson elliptic flow in non-central Pb-Pb collisions at $\sqrt{s_{\mathrm{NN}}}$ = 2.76 TeV. Phys. Rev. Lett..

[32] Abelev B.B., Ajaz M., Khan K.H., Sleymanov M.K., Zaman A. (2014). Azimuthal anisotropy of D meson production in Pb-Pb collisions at $\sqrt{s_{\mathrm{NN}}}$ = 2.76 TeV. Phys. Rev. C.

[33] Adam J., Adamová D., Aggarwal M.M., Rinella G.A., Agnello M., Agrawal N., Ahammed Z., Ahn S.U., Aimo I., Aiola S. (2015). Centrality dependence of high-p_T_ D meson suppression in Pb-Pb collisions at $\sqrt{s_{\mathrm{NN}}}$ = 2.76 TeV. J. High Energy Phys..

[34] Sirunyan A.M., Tumasyan A., Adam W., Ambrogi F., Asilar E., Bergauer T., Brandstetter J., Brondolin E., Dragicevic M., Erö J. (2018). Measurement of prompt and nonprompt charmonium suppression in PbPb collisions at 5.02 TeV. Eur. Phys. J. C.

[35] Tumasyan A., Adam W., Andrejkovic J.W., Bergauer T., Chatterjee S., Damanakis K., Dragicevic M., Del Valle A.E., Hussain P.S., Jeitler M. (2023). Measurements of the azimuthal anisotropy of charmonia in PbPb collisions at $\sqrt{s_{\mathrm{NN}}}$ = 5.02 TeV. J. High Energy Phys..

[36] Bernhard J.E., Moreland J.S., Bass S.A., Liu J., Heinz U. (2016). Applying Bayesian parameter estimation to relativistic heavy-ion collisions: Simultaneous characterization of the initial state and quark-gluon plasma medium. Phys. Rev. C.

[37] Auvinen J., Bernhard J.E., Bass S.A., Karpenko I. (2018). Investigating the collision energy dependence of *η*/s in the beam energy scan at the BNL Relativistic Heavy Ion Collider using Bayesian statistics. Phys. Rev. C.

[38] Novak J., Novak K., Pratt S., Vredevoogd J., Coleman-Smith C., Wolpert R. (2014). Determining Fundamental Properties of Matter Created in Ultrarelativistic Heavy-Ion Collisions. Phys. Rev. C.

[39] Pratt S., Sangaline E., Sorensen P., Wang H. (2015). Constraining the Eq. of State of Super-Hadronic Matter from Heavy-Ion Collisions. Phys. Rev. Lett..

[40] Xu Y., Bernhard J.E., Bass S.A., Nahrgang M., Cao S. (2018). Data-driven analysis for the temperature and momentum dependence of the heavy-quark diffusion coefficient in relativistic heavy-ion collisions. Phys. Rev. C.

[41] Cacciari M., Greco M., Nason P. (1998). The p(T) spectrum in heavy-flavour hadroproduction. J. High Energy Phys..

[42] Cacciari M., Frixione S., Nason P. (2001). The p(T) spectrum in heavy-flavor photoproduction. J. High Energy Phys..

[43] Ball R.D., Bertone V., Carrazza S., Deans C.S., Del Debbio L., Forte S., Guffanti A., Hartland N.P., Latorre J.I., Rojo J. (2015). Parton distributions for the LHC Run II. J. High Energy Phys..

[44] Eskola K.J., Paukkunen H., Salgado C.A. (2009). EPS09: A New Generation of NLO and LO Nuclear Parton Distribution Functions. J. High Energy Phys..

[45] Yang M., Zheng S., Tong B., Zhao J., Ouyang W., Zhou K., Chen B. (2023). Bottom energy loss and nonprompt J/*ψ* production in relativistic heavy ion collisions. Phys. Rev. C.

[46] Cao S., Qin G.Y., Bass S.A. (2013). Heavy-quark dynamics and hadronization in ultrarelativistic heavy-ion collisions: Collisional versus radiative energy loss. Phys. Rev. C.

[47] Greco V., Ko C.M., Rapp R. (2004). Quark coalescence for charmed mesons in ultrarelativistic heavy ion collisions. Phys. Lett. B.

[48] Schenke B., Jeon S., Gale C. (2011). Elliptic and triangular flow in event-by-event (3+1)D viscous hydrodynamics. Phys. Rev. Lett..

[49] Schenke B., Jeon S., Gale C. (2010). (3+1)D hydrodynamic simulation of relativistic heavy-ion collisions. Phys. Rev. C.

[50] Altenkort L., Eller A.M., Kaczmarek O., Mazur L., Moore G.D., Shu H.T. (2021). Heavy quark momentum diffusion from the lattice using gradient flow. Phys. Rev. D.

[51] Brambilla N., Leino V., Petreczky P., Vairo A. (2020). Lattice QCD constraints on the heavy quark diffusion coefficient. Phys. Rev. D.

[52] Liu S.Y.F., Rapp R. (2020). Spectral and transport properties of quark–gluon plasma in a nonperturbative approach. Eur. Phys. J. A.

[53] Scardina F., Das S.K., Minissale V., Plumari S., Greco V. (2017). Estimating the charm quark diffusion coefficient and thermalization time from D meson spectra at energies available at the BNL Relativistic Heavy Ion Collider and the CERN Large Hadron Collider. Phys. Rev. C.

[54] ALICE collaboration (2022). Prompt D^0^, D^+^, and D^*+^ production in Pb–Pb collisions at $\sqrt{s_{\mathrm{NN}}}$ = 5.02 TeV. J. High Energy Phys..

[55] Altenkort L., de la Cruz D., Kaczmarek O., Larsen R., Moore G.D., Mukherjee S., Petreczky P., Shu H.T., Stendebach S. (2023). Quark Mass Dependence of Heavy Quark Diffusion Coefficient from Lattice QCD. arXiv.

[56] Casalderrey-Solana J., Teaney D. (2006). Heavy quark diffusion in strongly coupled N=4 Yang-Mills. Phys. Rev. D.

[57] Andreev O. (2018). Drag Force on Heavy Quarks and Spatial String Tension. Mod. Phys. Lett. A.

[58] Combridge B.L. (1979). Associated Production of Heavy Flavor States in p p and anti-p p Interactions: Some QCD Estimates. Nucl. Phys. B.

[59] Caron-Huot S., Moore G.D. (2008). Heavy quark diffusion in perturbative QCD at next-to-leading order. Phys. Rev. Lett..

[60] Caron-Huot S., Moore G.D. (2008). Heavy quark diffusion in QCD and N=4 SYM at next-to-leading order. J. High Energy Phys..

[61] Das S.K., Scardina F., Plumari S., Greco V. (2015). Toward a solution to the *R*_AA_ and *v*_2_ puzzle for heavy quarks. Phys. Lett. B.

[62] Baier R., Dokshitzer Y.L., Mueller A.H., Peigne S., Schiff D. (1997). Radiative energy loss and p(T) broadening of high-energy partons in nuclei. Nucl. Phys. B.

[63] He M., Fries R.J., Rapp R. (2013). **D**_s_-Meson as Quantitative Probe of Diffusion and Hadronization in Nuclear Collisions. Phys. Rev. Lett..

[64] Barnard J., Dawe E.N., Dolan M.J., Rajcic N. (2017). Parton Shower Uncertainties in Jet Substructure Analyses with Deep Neural Networks. Phys. Rev. D.

